# Evaluation of ctDNA in the Prediction of Response to Neoadjuvant Therapy and Prognosis in Locally Advanced Rectal Cancer Patients: A Prospective Study

**DOI:** 10.3390/ph16030427

**Published:** 2023-03-10

**Authors:** Marina Morais, Telma Fonseca, Diogo Melo-Pinto, Isabel Prieto, Ana Teresa Vilares, Ana Luísa Duarte, Patrícia Leitão, Luís Cirnes, José Carlos Machado, Silvestre Carneiro

**Affiliations:** 1 Department of Surgery, Hospital Pedro Hispano, 4464-513 Matosinhos, Portugal; 2 Department of Surgery, Centro Hospitalar Universitário de São João EPE, 4200-319 Porto, Portugal; 3 Department of Surgery, Hospital La Paz, 28046 Madrid, Spain; 4 Department of Radiology, Centro Hospitalar Universitário de São João EPE, 4200-319 Porto, Portugal; 5 Department of Radiology, Hospital Pedro Hispano, 4464-513 Matosinhos, Portugal; 6 Diagnostics Group, Institute of Molecular Pathology and Immunology of Porto University, 4200-135 Porto, Portugal

**Keywords:** ctDNA, next-generation sequencing, locally advanced rectal cancer, neoadjuvant chemoradiation, prediction of response, disease-free survival

## Abstract

“Watch and wait” is becoming a common treatment option for patients with locally advanced rectal cancer (LARC) submitted to neoadjuvant treatment. However, currently, no clinical modality has an acceptable accuracy for predicting pathological complete response (pCR). The aim of this study was to assess the clinical utility of circulating tumor DNA (ctDNA) in predicting the response and prognosis in these patients. We prospectively enrolled a cohort of three Iberian centers between January 2020 and December 2021 and performed an analysis on the association of ctDNA with the main response outcomes and disease-free survival (DFS). The rate of pCR in the total sample was 15.3%. A total of 24 plasma samples from 18 patients were analyzed by next-generation sequencing. At baseline, mutations were detected in 38.9%, with the most common being *TP53* and *KRAS*. Combination of either positive magnetic resonance imaging (MRI) extramural venous invasion (mrEMVI) and ctDNA increased the risk of poor response (*p* = 0.021). Also, patients with two mutations vs. those with fewer than two mutations had a worse DFS (*p* = 0.005). Although these results should be read carefully due to sample size, this study suggests that baseline ctDNA combined with mrEMVI could potentially help to predict the response and baseline ctDNA number of mutations might allow the discrimination of groups with different DFS. Further studies are needed to clarify the role of ctDNA as an independent tool in the selection and management of LARC patients.

## 1. Introduction

Colorectal cancer is a major health burden worldwide, with 1.88 million new cases and 0.92 million new deaths reported in 2020 [1]. Rectal cancer represents one third of the cancers of the large intestine. Preoperative chemoradiotherapy (nCRT) followed by radical surgery is the standard of care for patients with locally advanced rectal cancer (LARC) due to improvement in local recurrence rates [2], downstaging of the tumor [3], better sphincter preservation [4] and a safer closer distal margin at resection [5,6]. However, novel approaches are emerging, such as total neoadjuvant therapy (TNT) followed by surgery, in order to improve these outcomes [7], treating occult micrometastases several months earlier, increasing treatment compliance, enhancing the efficacy of chemotherapy and ultimately improving survival [8].

A pathological complete response (pCR) to neoadjuvant treatment, in which no viable tumor cells are present in the surgical specimen, is achieved in 15–20% of patients [9]. These patients benefit from improved local and distant disease control and could potentially avoid the complications of surgery [10]. Surgery has been associated with long-term sequelae, including urinary and sexual dysfunctions, fecal incontinence, temporary or permanent stomas and immediate post-operative risks, such as bleeding, infection and anastomotic leak [11]. The elderly population, in which there is a higher prevalence of mismatch repair-deficient cancers with microsatellite instability, larger and locally invasive disease and lower lymph node metastasis, is even more prone to severe post-operative complications, especially those with pT4 disease [12]. Novel approaches could benefit these patients. The “watch and wait” approach was popularized by Habr-Gama et al. [10] in 2004 for patients who achieve complete clinical response (cCCR). These patients do not receive any surgery and are maintained under close surveillance. However, to this date, none of the clinical tools available, including endorectal ultrasound or magnetic resonance imaging (MRI), has an acceptable accuracy for predicting pCR [13].

A promising biomarker of pCR is the circulating tumor DNA (ctDNA) collected in liquid biopsies, which is secreted from cancer cells into the peripheral blood as a result of cell apoptosis, necrosis or via exosomes [14,15] and represents a small fraction (<1% in some studies) of the circulating free DNA [16]. This biomarker has shown application in monitoring treatment response, cancer recurrence and drug resistance in colorectal cancer patients [17,18,19]. Due to its short turnover time, ctDNA may provide a real-time information about the disease [20]. Some limitations have been attributed to ctDNA, such as the generally low concentrations extracted from plasma, background noise from non-tumor cells or induced by inflammation and trauma [21] and exacerbation of theses by improper pre-analytical handling [22]. Nevertheless, it was reported that ctDNA was a more sensitive method than CEA or radiological imaging in detecting disease progression [17,23,24] and that ctDNA might circumvent the limitations of tissue biopsies, such as invasiveness and not representing the whole heterogeneity of the disease [25]. The next-generation sequencing (NGS) technique uses commercially available kits and is easy to implement in a laboratory. It has the advantage of allowing the characterization of different genes in a single run using a smaller quantity of DNA for obtaining a large amount of molecular data and for its good sensitivity (0.1%) in comparison with some of the most common polymerase chain reaction (PCR)-based methodologies.

As examined before in a systematic review [26], results are still very inconsistent across studies about which mutations, methylations, methods or timings are more accurate for predicting response and prognosis. The aim of this study was to add some information to the literature on the clinical utility of baseline and serial ctDNA analysis in predicting response to neoadjuvant treatment and prognosis in LARC patients.

## 2. Results

### 2.1. Study Enrollment

**Figure 1:**
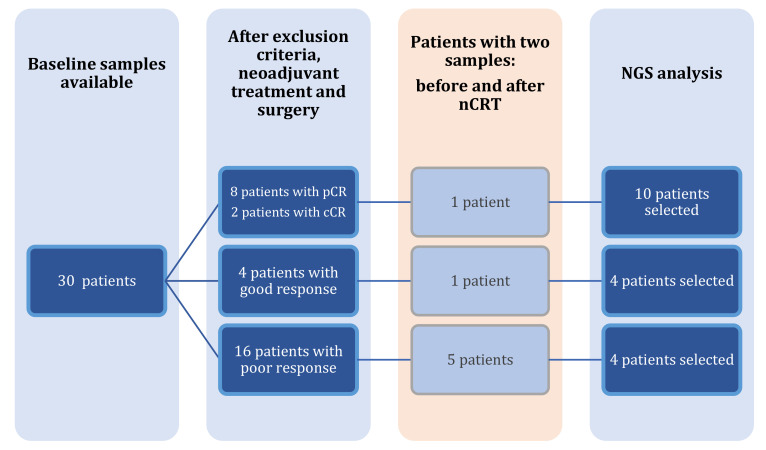
Study flowchart of selection of patients for NGS analysis.

We prospectively enrolled 30 patients with LARC who met inclusion criteria, after excluding patients who did not accept to participate in the study (*n* = 6), in which disease progressed (*n* = 9) or with missing baseline samples (*n* = 7). The rate of pCR in the total sample was 15.3% (8/52 patients). Figure 1 shows the flowchart of patient selection for NGS analysis. After obtaining response outcomes, the patients were divided into groups of response to therapy for further analysis. Eight patients achieved pCR, two patients achieved cCR, four patients had a good response and sixteen patients presented a poor response. From these, only seven patients had two samples collected before and after neoadjuvant treatment. All patients with a complete or good response were selected for NGS analysis: however, only four patients with baseline and post-treatment samples from the poor response group were chosen for the analysis as controls for the main outcomes of the study (complete and good response) due to budget restrictions. We ended up with a cohort of 18 patients.

### 2.2. Clinicopathological Features

The median age of patients was 65 years old (38–86). Eight patients (44.4%) were males and ten (55.6%) were females. The median distance from the anal verge was 6 cm (2–12). Pretreatment CEA median levels were 2.6 ng/mL and pretreatment mrEMVI was positive in nine (50%) of patients. Fourteen patients (77.8%) were diagnosed as clinical stage III and three patients (16.7%) as clinical stage II; two patients (11.1%) had clinical T4 and sixteen (88.9%) clinical T3. Twelve patients (66.7%) received standard nCRT and six received short-course radiotherapy and neoadjuvant chemotherapy. After preoperative therapy, two patients achieved cCR (ycT0N0M0), nine patients had a good response and five presented with a poor response in reevaluation MRI. Twelve patients (66.7%) were submitted to anterior rectal resection, whereas four (22.2%) had an abdomino-perineal resection performed. The median interval from completing preoperative therapy to surgery was 10 weeks (8–26). After surgery, eight patients had pCR and four patients achieved good response (TRG 1). One patient had ypT1 stage, one had ypT2 stage, six presented with ypT3 stage and three patients had positive ypN post-operatively. Furthermore, two patients (12.5%) presented mucinous histology, three (16.7%) had venous invasion and one (5.6%) had perineural invasion. Five patients (27.8%) were treated with adjuvant chemotherapy and at a median follow-up of 14 months, we observed three (16.7%) systemic recurrences. Table 1 shows the clinico-pathological features of this sample. 

### 2.3. Detection of Somatic Mutations in Plasma

**Figure 2:**
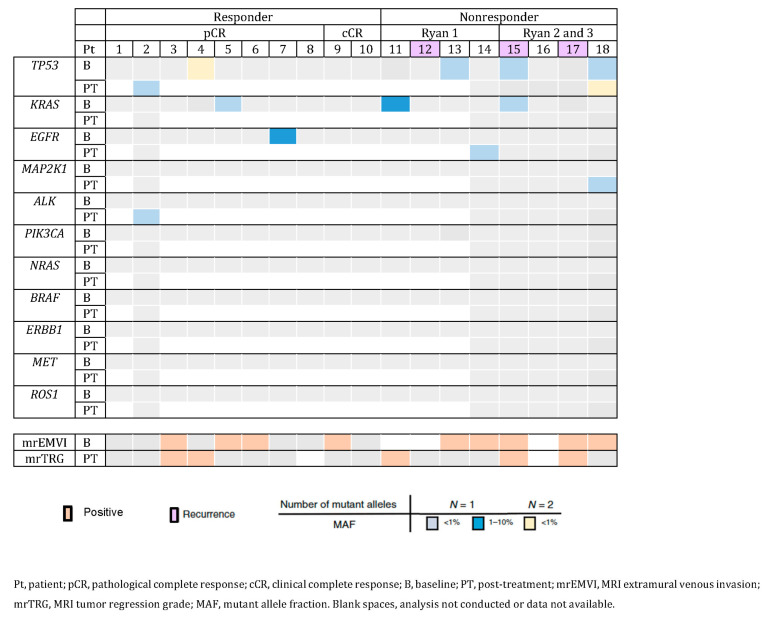
Genomic landscape of the mutations detected in plasma and imaging data.

A total of 24 plasma samples from 18 patients were analyzed by NGS. One or more somatic mutations (mutant alleles) were detected in seven (38.9%) patients at baseline and in four out of six (66.7%) patients after preoperative treatment. One or more somatic mutations in TP53, KRAS and EGFR genes were detected in four (22.2%), three (16.7%) and one (5.5%) patient at baseline, respectively. In the post-treatment analysis, we found mutations of TP53 in three patients and mutations in EGFR, MAP2K1 and ALK in one patient each. Two post-treatment mutations were acquired, not detectable at baseline, and in one patient, a TP53 mutation present at post-treatment samples was already present at baseline. These results are shown in Figure 2. The distribution of baseline mrEMVI and post-treatment MRI reevaluation results are also shown in this Figure.

Patients were classified into four groups, as shown in Figure 2: pCR (*n* = 8), cCR (*n* = 2), Ryan 1 (*n* = 4) and Ryan 2 and 3 (*n* = 4).

In the pCR group, one patient had TP53 and ALK mutations with MAF < 1% that appeared after treatment (the only patient of the pCR group with post-treatment samples collected), one patient had two mutations of TP53 with a MAF < 1% before treatment, one patient had a KRAS mutation with MAF < 1% and one patient had an EGFR mutation with MAF between 1 and 10%. No mutations were found in the other patients, as no mutations were found for the cCR group, also with no samples collected post-treatment.

For non-responders, in the Ryan 1 group, only one patient had both samples collected, and in this patient, a new mutation in EGFR appeared after treatment. In one patient a KRAS mutation with a MAF between 1 and 10% was found at baseline, and in another patient a TP53 mutation with MAF < 1% was found. For the Ryan 2 and 3 group, both samples were collected in the four patients selected. One patient had TP53 and KRAS mutations at baseline with MAF < 1%, and another patient presented with a TP53 mutation with MAF < 1% at baseline and with two mutations in TP53 and one mutation in MAP2K1 with MAF < 1% after treatment. In the other two patients, no mutations were found.

### 2.4. Association between Response to Preoperative Therapy and ctDNA

**Figure 3:**
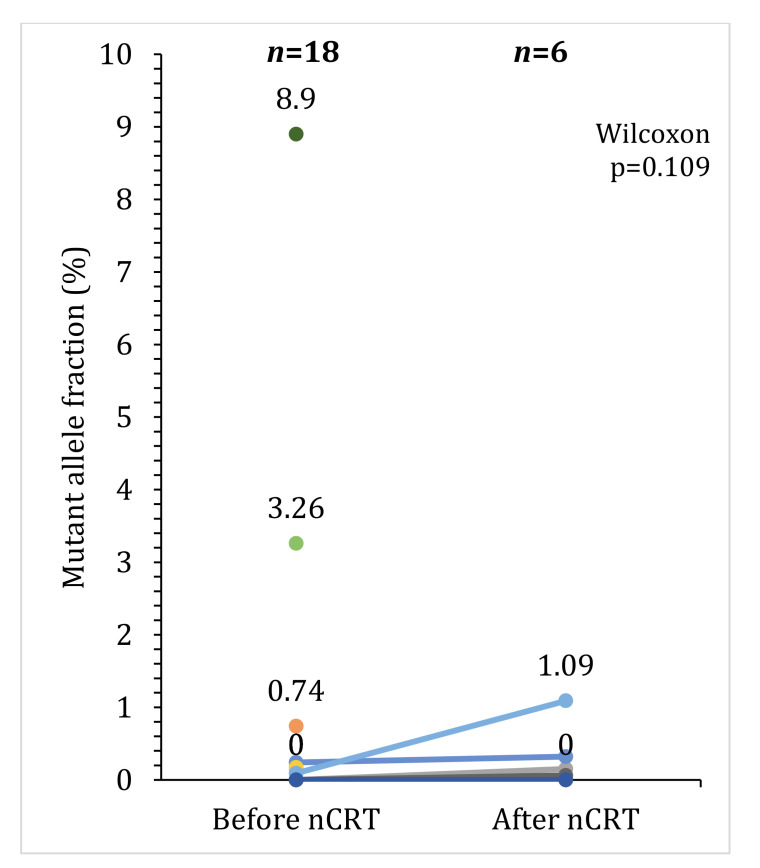
Mutant allele fractions in baseline and post-treatment samples and changes between the two timepoints.

As shown in Table 2, in our sample nonresponders and poor responders had a higher frequency of positive baseline ctDNA and a lower frequency of post-treatment ctDNA, although no significant association was observed. The MAF distribution is presented in Figure 3, where we can observe that most patients at baseline presented a MAF < 1% and in the six patients with two blood samples collected (pre- and post-treatment), MAF has non-significantly (Wilcoxon test, *p* = 0.109) increased between the two timepoints.

In Figure 2, we showed that all nonresponders with mrEMVI available were mrEMVI positive, whereas for responders this variable is more scattered. In what concerns mrTRG, we observed that non imagiological responders (mrTRG3-4) were also scattered across the groups (responders vs. nonresponders).

In Table 2, we showed that baseline mrEMVI could discriminate nonresponses from complete responders (*p* = 0.044). We then created a variable combining patients with either positive mrEMVI or positive baseline ctDNA and found that this variable was associated with a higher risk of poor response. Due to our small sample, a multivariable logistic regression was not attained. No other parameter could predict response, namely cTN stage, neoadjuvant therapy regimen, interval from therapy to surgery or mrTRG. Furthermore, in additional analysis not presented in the tables, ctDNA was not significantly associated with any of the other clinico-pathological features of the patients.

### 2.5. Association between Disease-Free Survival and ctDNA

We also evaluated the value of ctDNA in disease-free survival. With a median follow-up time of 14 (11–30) months, no patient died and a total of three (16.7%) recurrences, as lung metastases, were registered at a median of 12 months. As seen in Figure 2, patients with numbers 12, 15 and 17 presented with recurrence and were nonresponders. They all were mrEMVI positive and two of them were imagiological poor responders (mrTRG 3-5). In two of them, no mutations were found; in the other patient, two mutations were found (*TP53* and *KRAS*).

**Figure 4:**
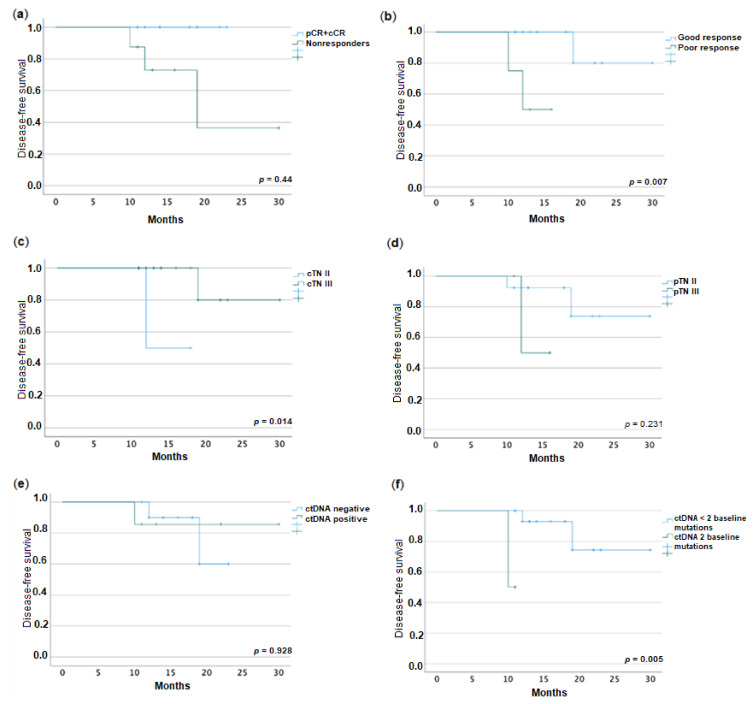
Kaplan–Meier curves of disease-free survival. (**a**) Responders vs. nonresponders; (**b**) Good response vs. poor response; (**c**) Clinical TN stage II vs. III; (**d**) Pathological TN stage II vs. III; (**e**) ctDNA negative vs. positive; (**f**) ctDNA baseline mutations <2 vs. >2.

Kaplan–Meier curves, shown in Figure 4, revealed differences in DFS between responders and nonresponders (*p* = 0.004), good responders and poor responders (*p* = 0.007), and clinical stages (*p* = 0.014). We registered no impact of pathological stage on prognosis. No difference in DFS was found for positive baseline or post-treatment ctDNA vs. negative. However, when we accounted for the number of mutations present, those patients with two mutations vs. fewer than two mutations had a worse DFS (*p* = 0.005). No other clinico-pathological factors studied had an impact on prognosis; we did not proceed to search for independent factors of prognosis due to the size of our sample.

## 3. Discussion

Several studies have sought to identify clinically useful predictors of pCR after preoperative therapy for patients with LARC [27]. Some parameters have been reported as possible predictors, including baseline CEA levels, distance of the tumor from the anal verge, tumor size, clinical lymph node metastasis and interval between treatment and surgery [27,28,29]. Habr-Gama et al., suggested that strict clinical and endoscopic findings of patients with cCR could be useful to select patients for “watch and wait” strategies [30]. However, endoscopic evaluation or MRI evaluation for the response after treatment have low accuracy for pCR [13], with a sensitivity of only about 25% [31]. Furthermore, it has been reported that local regrowth after cCR was about 25% to 50% [32,33], whereas after pCR it was only about 3% [9], with a higher rate of distant metastases after local regrowth (36% vs. 1%) [34]. These data call for an improvement of the methods currently used for predicting pCR.

The systemic biomarker ctDNA is readily available through liquid biopsy and has been shown to detect micrometastasis or minimal residual disease earlier than imaging [35]. Even though some studies reported an association between survival outcomes and ctDNA [17,19,36,37,38,39,40], fewer studies have shown an association with pCR [37,41]. In our study, we intended to understand whether baseline ctDNA (evaluated by NGS available in our laboratory) could help predict response to nCRT in LARC patients. Although we had a small sample limited by budget, we opted to also study the available post-treatment samples, as Murahashi et al. (*n* = 85) had previously shown that dynamic changes in ctDNA performed better than baseline ctDNA in predicting response [37]. The baseline ctDNA was positive in 38.9% of cases, and this rate varies largely in the literature, going from 15 to 77% [42]. Our results showed that baseline ctDNA was not associated with pCR or good response as previously found in other works [36,37]. However, nonresponders or poor responders most frequently had positive baseline ctDNA versus complete or good responders, which shows a tendency already observed in previous studies [38,43]. The lack of correlation between baseline ctDNA and pathological response might reflect the heterogeneity of sensitivity from the tumor to neoadjuvant treatment. Unlike Pazdirek et al. [40] and Wang et al. [33], in which ctDNA was strongly eliminated from plasma from baseline to post-treatment samples (probably due to tumor shrinkage and less ctDNA available from enzymatic digestion induced by radiation damage [44]), in our study we observed that some mutations appeared in the post-treatment sample that were not present initially, although the MAF did not change significantly and could not predict outcomes. This was probably related to the low number of post-treatment analyses or the limited sensitivity of NGS for identifying pCR, as reported by Roesel et al. [45].

The most common genes mutated in our sample were *TP53* and *KRAS*. TP53 mutation is observed in 50–75% of CRCs. p53 plays a crucial role in regulating DNA repair and apoptosis in response to radiation, and *TP53* mutations are reported to decrease radiation-induced apoptosis in several cancers [46]. Tumors with *TP53* mutations tend to accumulate through neoadjuvant treatment [47]. This suggests that chemoradiation therapy provides a selective pressure for the expansion of *TP53*-mutant cells in residual tumors [47]; as could happen with other mutations that appeared later in our sample. A meta-analysis of 1830 cases with low wild-type *TP53* status has shown an association with pathological response in LARC patients [48]. Although we could not demonstrate which mutation was more impactful for a good response, *TP53* mutation was the most common mutation among our patients. Furthermore, in the literature miR-34a has been reported as one of the most relevant downstream effectors of p53, which blocks the IL-6R/STAT3/miR-34 feedback loop resulting in inhibition of tumor progression in rectal cancer [49]. miR-34a might therefore represent another important biomarker and therapeutic target in rectal cancer.

Also, *KRAS* mutation, the second most common in our sample, belongs to the family of genes most frequently present in human cancers [50]. *KRAS* leads to an epidermal-growth-factor-receptor-independent disturbance of the RAS/RAF/MAPK pathway, which regulates proliferation and survival in rectal cancer [51]. *KRAS* mutation and combined *KRAS/TP53* mutations have been previously independently associated with poor responses after neoadjuvant treatment [52,53], although other reports found no correlation with response [54]. Furthermore, KRAS mutations have been associated with a shorter survival in patients with MSI-low tumors. Therefore, the screening for defective DNA mismatch repair through immunohistochemistry (IHC) and/or microsatellite instability (MSI) tests should be performed, although there are still challenges due to the heterogeneity of MSI testing [55].

Previous studies have found an additional benefit for combining imaging features, such as mrTRG and ctDNA, in the prediction of response [33,56]. In our analysis, although mrTRG and none of the other clinical factors were associated with response or ctDNA, we found that baseline positive mrEMVI could predict nonresponders. Also, after combining baseline positive ctDNA and mrEMVI, when at least one of these variables were present the risk of poor response increased. An association between ctDNA and mrEMVI has been observed before in Zhou et al.’s work [38]. This could raise our awareness of the fact that not one but many factors might have to be taken into account to predict response, as many pathways might be involved in the response to chemotherapy. As an indicator that can be assessed by radiologists, although still with a variable inter-rate kappa reliability, the clinical value of mrEMVI and the potential clinical significance of its correlation with ctDNA in predicting response deserve further investigation.

With a median of 14 months of follow-up, tumor progression with distant metastases was registered in 3 out of 18 patients. As previously demonstrated in the literature [57] and also in our study, complete and good responses to therapy improved disease-free survival. Interestingly, patients with cTN II had a worse DFS than those with cTN III. This could possibly be explained by Kim et al.’s [58] so-called “survival paradox”, in which the authors attributed a hypothetical higher biological aggressiveness to pT4N0 colon carcinomas. Besides these factors, no other clinico-pathological factors, such as distance to anal verge, mrEMVI, mrTGR, CEA, pTN, mucinous histology, or vascular or perineural invasion were associated with DFS. As for the marker of study, baseline ctDNA or MAF did not predict recurrence, as seen before [38]. When we studied a small group of patients harboring two mutations instead of one, these could differentiate a group with worse DFS. However, Ji et al. [59] found that lower tumor mutation burden at baseline was associated with worse DFS, for these tumors with higher burden might have a higher immune activity and be more immunogenic, which leads to greater tumor shrinkage. Nevertheless, more work is still needed to understand if different signatures might be associated with different outcomes. Also, the dynamic surveillance of ctDNA throughout treatment has shown to be a more reliable method to predict a higher risk of recurrence [26], which was not possible in this study. The exploration of this method could help clinicians direct the high-risk patients to more intensified chemotherapy regimens.

Our study has several limitations. First, due to the small number of recruited patients and samples (due to logistic problems and non-acceptance by some patients) and the short follow-up period, there were too few events to correct for potential confounding factors and to give enough power to ctDNA to come up as independent factor of pCR and disease-free survival. Second, patients underwent different preoperative therapy regimens based on different risk profiles. Third, tumor biopsy sequencing was not performed in our study. Nevertheless, some mutations detected on ctDNA might not be detected in tumor biopsies, which reflects the tumor evolution and heterogeneity not captured in small biopsy samples. Furthermore, the proportion of patients with positive ctDNA at baseline was low, which could be due to false-negative results related to quantitative (insufficient MAF content) rather than qualitative (lack of altered genes in the NGS panel) reasons. Technical improvement in the gene panel could perhaps increase the detection accuracy. A strength of this study relies on the fact that all samples were collected prospectively, which increases quality of samples and provides more reliable results.

## 4. Materials and Methods

### 4.1. Patients

This study prospectively enrolled a pilot cohort of patients with LARC from three Iberian centers (Pedro Hispano Hospital, Matosinhos; Centro Hospitalar Universitário de São João, Porto; and Hospital La Paz, Madrid) between January 2020 and December 2021. All patients were diagnosed with cT3-4 or N+ rectal adenocarcinoma (within 12 cm from anal verge) using the AJCC 8th edition TNM classification [60]. Inclusion criteria included age above 18, treatment with neoadjuvant therapy followed by radical surgery, no metastasis at diagnosis or previous cancers, Eastern Cooperative Oncology Group performance status of 0–2 and written informed consent to participate in the study for sample collection, gene sequencing and data analysis. Patients proposed to the “watch and wait” strategy were also included. This study was approved by the Institutional Review Boards of all Hospitals and was registered at ClinicalTrials.gov (NCT04319354).

The following clinico-pathological data were collected prospectively: age, sex, tumor location from anal verge, clinical stage, mrEMVI, neoadjuvant treatment regimen, MRI response, type of surgery, pathological stage, mucinous histology and venous and perineural invasions.

### 4.2. Treatment

Patients received either one of two neoadjuvant treatment regimens, according to multidisciplinary team decision, taking into account patient and tumor features and point in time of treatment proposal. Mostly in the beginning of the series, the treatment consisted of standard neoadjuvant chemoradiotherapy (nCRT) comprising a total dose of 50.4 Grey over 5 weeks and concurrent capecitabine or 5-fluorouracil. The second treatment consisted of short-course radiotherapy (SCRT), with 25 Gy administered over 1 week and followed by neoadjuvant chemotherapy with combinations of 5-fluouracil and folinic acid or capecitabine, plus oxaliplatin (TNT). After completing all neoadjuvant treatments, all patients were re-staged by rectal MRI and then proposed to surgery or the “watch and wait” approach.

### 4.3. Response Assessment

MRI assessment of tumor regression grade (mrTRG) was evaluated according to Mercury criteria by the radiologists [61]. mrTRG 1 was categorized as “complete regression”, mrTRG 2 as “good regression” and mrTRG 3 to 4 as “poor regression”.

pCR was defined as ypT0N0M0. Pathological response was assessed by the Ryan TRG system [62] as follows: Ryan 1, no viable cells or single cells or small groups of residual cancer cells; Ryan 2, residual cancer outgrown by fibrosis; and Ryan 3, significant fibrosis outgrown by cancer or no fibrosis with extensive residual cancer. Clinical complete response (cCR), a surrogate of pCR, was defined as ycT0N0M0, evaluated by clinical examination (digital rectal examination, endoscopy and MRI) in patients with no disease progression after the 12-month follow-up. Furthermore, for the analysis, the following groups were created: “responders” (cCR + pCR) vs. “nonresponders” (the remaining) and “good responders” (cCR + pCR + Ryan 1) vs. “poor responders” (Ryan 2 + Ryan 3). At the end of patient recruitment and after knowledge of these outcomes, the collected samples were then selected for further analysis of ctDNA.

### 4.4. Blood Sampling, Cell-Free DNA Isolation and Sequencing

Serum carcinoembryonic antigen (CEA) level was measured at baseline. For ctDNA analysis, blood samples were collected 1–2 weeks before initiation of preoperative therapy (baseline) and after treatment (just before surgery) for some patients. Blood samples were collected into BD Vacutainer® PPT™ plasma preparation tubes (2 × 5 mL) (BD Biosciences, Franklin Lakes, NJ, USA). Plasma was extracted by centrifugation at 1100 rcf for 10 min at room temperature and stored at −20 °C according to laboratory protocol. The samples were purified using PromegaInstrument Maxwell^®^ RSC (Promega Corporation, Madison, WI, USA). Libraries from cfDNA were prepared with Oncomine Lung cfDNA Assay (Thermo Fisher Scientific, Waltham, MA, USA), available in the laboratory for the most common mutations in rectal cancer. The cfDNA panel used in this study covered 11 genes (*ALK*, *BRAF*, *EGFR*, *ERBB2*, *KRAS*, *MAP2K1*, *MET*, *NRAS*, *PIK3CA*, *ROS1* and *TP53*), with more than 150 hotspots (SNVs and short indels). We tracked the mutations with the highest mutation allele frequency (MAF) in each patient [63]. Patients with detectable mutations were categorized “ctDNA positive”, and the ones with undetectable mutations were characterized as “ctDNA negative”.

### 4.5. Statistical Analysis

Statistics were performed using IBM SPSS Statistics 28.0.10 (IBM, Armonk, NY, USA). Fisher’s exact test, McNemar test and Mann–Whitney tests were used to compare the risk factors in the study with the main treatment response outcomes. The Wilcoxon test was used to compare median MAF between pre-and post-treatment samples. Survival analysis was conducted through the Kaplan–Meier log rank method to analyze the importance of ctDNA and other factors in disease-free survival (DFS), which was defined as time without recurrence since diagnosis until last follow-up. Values with *p* < 0.05 were considered statistically significant.

## 5. Conclusions

In conclusion, although these results should be read carefully due to sample size, this study suggests that baseline ctDNA combined with mrEMVI could potentially help predict response to nCRT in LARC patients. Additionally, baseline ctDNA number of mutations might allow the discrimination of groups with different disease-free survival times. Further studies with more patients, samples, timepoints and clinical data integrated with a large number of cancer-related genes are needed to clarify the role of ctDNA as an independent tool in the selection and management of LARC patients. The authors believe that the integration of the data from this study with data coming from other studies in future meta-analyses will likely be useful to provide a better guide for clinical practice by healthcare professionals.

## Figures and Tables

**Table 1:** Clinico-pathological features of the patients.

	***N***	**%**
**Age, years, median**	65 (38–86)	
**Sex**		
**Male**	8	44.4
**Female**	10	55.6
**BMI, kg/m^2^, median**	25.6 (16.9–30.7)	
**Smokers**	6	33.3%
**Tumor location from the anal verge, median cm**	6 (2–12)	
**Pretreatment CEA, median ng/mL**	2.6 (1–21)	
**Pretreatment positive ctDNA**	7	38.9
**Post-treatment positive ctDNA**	4	66.7
**Clinical T**		
**cT3**	16	88.9
**cT4**	2	11.1
**Clinical stage**		
**II**	3	16.7
**III**	14	77.8
**mrEMVI positive**	9	50.0
**Neoadjuvant treatment**		
**nCRT**	12	66.7
**SCRT+TNT**	6	33.3
**Interval from therapy to surgery, median weeks**	10 (8–26)	
**MRI response**		
**Complete (mrTRG1)**	2	11.1
**Good (mrTRG2)**	9	50.0
**Poor (mrTRG3-5)**	5	27.8
**Surgery**		
**Anterior resection**	12	66.7
**APR**	4	22.2
**Pathological T**		
**ypT0-2**	10	55.6
**ypT3-4**	6	33.3
**Pathological N**		
**ypN0**	13	72.2
**ypN1-2**	3	16.7
**Mucinous histology**	2	12.5
**Venous invasion**	3	16.7
**Perineural invasion**	1	5.6
**Adjuvant therapy**	5	27.8
**Follow-up (months)**	14 (11–30)	
**Recurrence (systemic)**	3	16.7

CEA, carcinoembryonic antigen; mrEMVI, MRI detected extramural vascular invasion; nCRT, neoadjuvant chemoradiotherapy; SCRT, short-course radiotherapy; TNT, total neoadjuvant therapy; MRI, magnetic resonance imaging; mrTRG, MRI tumor regression grade; APR, abdomino-perineal resection. Continuous variables presented as median (minimum–maximum range).

**Table 2:** Summary of the relationship between risk factors and main outcomes.

	Nonresponders vs. pCR + cCR	*p*	Poor Responders vs. Good Responders	*p*
**cTN III**	85.7% vs. 80.0%	1.000 ^a^	66.7% vs. 85.7%	0.465 ^a^
**mrEMVI+**	100% vs. 40.0%	**0.044 ^a^**	100% vs. 50.0%	0.229 ^a^
**NAC vs. nCRT**	37.5% vs. 30.0%	1.000 ^a^	50.0% vs. 28.6%	0.569 ^a^
**Interval therapy to surgery (weeks)**	11 vs. 9.5	0.161 ^b^	11 vs. 14	0.684 ^b^
**mrTRG 3-5**	42.9% vs. 22.2%	0.596 ^a^	66.7% vs. 23.1%	0.214 ^a^
**Baseline ctDNA+**	50.0% vs. 30.0%	0.630 ^a^	50% vs. 35.7%	1.000 ^a^
**Baseline MAF**	0.044 vs. 0.0	0.479 ^b^	0.044 vs. 0.0	0.904 ^b^
**Either mrEMVI or ctDNA+**	2.000 (0.260–15.381)	0.289 ^c^	1.667 (0.135–20.578)	**0.021 ^c^**
**Post-treatment ctDNA+**	60.0% vs. 100%	1.000 ^a^	50.0% vs. 100%	0.467 ^a^
**Post-treatment MAF**	0.057 vs. NA	0.776 ^a^	0.159 vs. 0.105	1.000 ^a^

pCR, pathological complete response; cCR, clinical complete response; cTN, clinical tumor node stage; mrEMVI, MRI extramural venous invasion; NAC, neoadjuvant chemotherapy; nCRT, neoadjuvant chemoradiotherapy; mrTRG, MRI tumor regression grade; ctDNA, circulating tumor DNA; MAF, mutant allele fraction; ^a^ Fisher exact test; ^b^ Mann–Whitney test; ^c^ McNemar test.

## Data Availability

Data are contained within the article.
